# Diet, iron biomarkers and oxidative stress in a representative sample of Mediterranean population

**DOI:** 10.1186/1475-2891-12-102

**Published:** 2013-07-16

**Authors:** Marta Romeu, Nuria Aranda, Montserrat Giralt, Blanca Ribot, Maria Rosa Nogues, Victoria Arija

**Affiliations:** 1 Pharmacology Unit, Faculty of Medicine and Health Sciences, Universitat Rovira i Virgili, Sant Llorenç 21,43201 Catalonia, Reus, Spain; 2 Nutrition and Public Health Unit, Faculty of Medicine and Health Sciences, Universitat Rovira i Virgili, Catalonia, Spain; 3 Institut de Investigació Sanitaria Pere Virgili (IISPV), Universitat Rovira i Virgili, Catalonia, Spain; 4 Institut de Investigació en Atenció Primària Jordi Gol i Gorina, Tarragona, Spain

**Keywords:** Diet, Antioxidant status, Oxidative stress, Iron, General population

## Abstract

**Background:**

The consumption pattern characterized by high consumption of vegetables, fruit, fish, olive oil and red wine has been associated with improvements in the total antioxidant capacity of individuals and reduced incidence of diseases related to oxidation. Also, high body iron levels may contribute to increase the oxidative stress by the generation of reactive oxygen species. The objective of this study is to analyze the relationship between antioxidant and pro-oxidant factors obtained from the diet and iron biomarkers on lipoprotein oxidation and total antioxidant capacity in a representative sample of the Mediterranean population.

**Methods:**

Cross-sectional prospective study, carried out with 815 randomly selected subjects (425 women and 390 men). Dietary assessment (3-day food records), iron biomarkers (serum ferritin, serum iron and transferrin saturation), biochemical markers of lipoperoxidation (TBARS), antioxidant capacity (ORAC) and CRP (C-Reactive Protein) were determined. Multiple Linear Regression (MLR) models were applied to analyze the association between diet factors and iron biomarkers on TBARS and ORAC levels.

**Results:**

We observed that lipoperoxidation measured by TBARS increased by age but no differences were observed by sex. Antioxidant capacity measured by ORAC is independent of age and sex. In general, increasing age, tobacco, heme iron intake from meat and fish and transferrin saturation were independently and positively associated with TBARS, while non-heme iron was negatively associated. Vegetables, vitamin C intake and serum ferritin were positively associated with ORAC, whereas saturated fatty acids and meat intake were negatively associated.

**Conclusions:**

In our general population, we observed that oxidative stress is related to aging, but antioxidant capacity is not. The highest intake of dietary non-heme iron, vegetables and vitamin C intake exerts a protective effect against oxidation while the highest intake of dietary heme iron from meat and fish and saturated fatty acids are associated with increased oxidative stress. High levels of circulating iron measured by transferrin saturation are associated with increased oxidative stress in women however its association with the higher levels of serum ferritin is controversial.

## Background

There is a pattern of consumption that is characterized by the high consumption of vegetables, fruit, fish, olive oil as the main source of fat, and the regular intake of red wine. This type of diet is rich in antioxidants and has been associated with improvements in the total antioxidant capacity of individuals [1] and reduced incidence of cardiovascular disease, cancer and other diseases related to oxidation [2], as well as the lower prevalence of overall mortality [3]. However, in some studies the increase in antioxidant capacity was not accompanied by a decrease in the susceptibility of lipoproteins to oxidative stress [4].

The regular daily diet of the Mediterranean population contains between 10 and 20 mg of iron in the form of heme and non-heme iron. About 40% of the iron from meat and fish is heme iron [5] which has high bioavailability and some authors suggest that it may act as a pro-oxidant factor [6]. Moreover, dietary factors could modify the bioavailability of non-heme iron [5]. In general, increased body iron may contribute to increased oxidative stress, since this is a transition metal that contributes to the generation of reactive oxygen species. Transition metals can promote lipid peroxidation in two ways: 1) by catalyzing the formation of oxygen free radical species capable of initiating lipid peroxidation and, 2) by catalyzing the decomposition of preformed lipid peroxides to propagate lipid peroxidation. However, the expected level of oxidative stress in a general Western population sample remains unknown; therefore, it is impossible to determine whether an increase in iron intake through diet alone leads to increased oxidative stress in healthy individuals [7].

Until now, most studies examining the relationship between iron consumption and oxidative stress have been in the form of clinical trials and studies of non-healthy patients or iron supplementation, but to our knowledge no studies have assessed the effect of the main dietary factors related to iron absorption and oxidation on oxidative stress in a representative sample of the Mediterranean population.

The objective of this study is to analyze the relationship between antioxidant and pro-oxidant factors obtained from the diet and iron biomarkers on lipoprotein oxidation and total antioxidant capacity in a representative sample of the Mediterranean population.

## Methods

### Subjects and study design

This cross-sectional and prospective study took place between 2005 and 2007. A representative and age-stratified sample of 815 individuals (425 women and 390 men) was randomly selected from the population registers of the town councils of three villages in the northeastern Mediterranean region of Spain (mean age, 42.5 years; range, 18–77 years). Inclusion criteria consisted of being Caucasian and 18 years or older. The criteria for exclusion were pregnancy, breastfeeding, neurological pathologies, cognitive retardation, and serious or chronic systemic disease. After project approval (Ethics Committee, Hospital Universitari Sant Joan de Reus, Universitat Rovira i Virgili) all the individuals signed an informed consent form in keeping with the requirements of the Helsinki Declaration.

All individuals underwent a clinical interview and data on lifestyle variables were collected (Table 1).

**Table 1:** General characteristics, antioxidant and pro-oxidant diet factors and iron biomarkers of the representative sample

	**All (n=815)**	**Men**^***(1) ***^**(n=390)**	**Women**^***(2) ***^**(n=425)**	***p-value ***^***(1–2)***^
**Age (years) **^**(a)**^	41(30–54)	42(30.75-54)	40(29–53)	0.877
**Age-group (years) %**				
group I (18–29)	24.3(18.3-30.2)	23.3(14.6-31.9)	25.2(16.9-33.4)	0.595
group II (30–44)	34.6(29.05-40.1)	36.4(28.5-44.3)	32.9(25.1-40.7)	0.333
group III (45–64)	29(23.2-34.8)	28.7(20.3-37.1)	29.2(21.2-37.2)	0.946
group IV (65–75)	12.1(5.7-18.5)	11.5(2.2-20.8)	12.7(3.8-21.6)	0.687
**BMI (kg/m**^**2**^**)**	27.1(26.6-27.3)	27.4(26.9-27.8)	26.7(26.1-27.2)	0.058
Underweight (BMI<18.5), %	1.4(0.6-2.2)	1.1(0.03-2.2)	1.7(0.4-2.9)	0.651
Normal (BMI: 18.5-25), %	37.3(33.8-40.7)	30(25.3-34.7)	43.9(39.1-48.7)	<0.001
Overweight (BMI 25–30), %	36.8(33.4-40.2)	43.3(38.2-48.4)	30.8(26.3-35.3)	<0.001
Obesity (BMI>30), %	24.5(21.4-27.5)	25.6(21.1-30.1)	23.6(19.4-27.7)	0.566
**Lifestyle**				
Alcohol drinkers (%)	41.7(38.3-45.1)	64.1(59.3-68.8)	21.2(17.3-25.1)	<0.001
Smokers (%)	34.2(30.9-37.4)	39.2(34.3-44)	29.6(25.2-33.9)	0.002
Habitual physical activity (%)	47.6(44.1-51)	53.6(48.6-58.5)	42.1(37.4-46.7)	0.001
**Education level (%)**				
None	3.2(−3.4-9.8)	2.3(−7.5-12.1)	4(−5.3-13.3)	0.240
Primary school	49.8(44.9-54.6)	50(42.9-57)	49.6(42.8-56.3)	0.922
High school	34.4(28.8-39.9)	35.9(27.9-43.8)	32.9(25.1-40.6)	0.415
University	12.6(6.2-19)	11.8(2.4-21.2)	13.4(46–22.2)	0.556
**Energy and antioxidant and pro-oxidant diet factors**				
Energy (kcal/d)	2195.1(2146.9-2243.1)	2567.1(2498.1-2636)	1865.8(1817.1-1914.4)	<0.001
SFA (g/d)	28.1(27.3-28.9)	32.3(31–33.6)	24.4(23.5-25.3)	<0.001
MUFA (g/d)	50.6(49.3-51.8)	58(56.2-59.9)	43.9(42.5-45.4)	<0.001
PUFA (g/d) ^(b)^	11.8(11.7-11.9)	14.05(13.9-14.2)	10.1(9.5-10.7)	<0.001
Vitamin C (μg/d) ^(b)^	85.5(85.3-85.7)	84.1(83.9-84.3)	86.8(86–87.5)	0.523
Vitamin E (μg/d) ^(b)^	11.4(11.2-11.5)	12.9(12.8-13.1)	10.1(10–10.3)	<0.001
β carotene (μg/d) ^(b)^	2217.8(2217.7-2218)	2052.8(2052.6-2053.1)	2375.1(2374.8-2375.4)	0.028
Retinol (μg/d) ^(a)^	236.2(153.6-353.3)	256.2(159.1-372.3)	215.0(147–320.4)	0.001
Non-heme iron (mg/d) ^(b)^	6.1(5.9-6.2)	6.8(6.6-6.9)	6.4(6.2-6.5)	0.036
Heme iron (mg/d) ^(b)^	2.9(2.8-3.1)	3.4(3.2-3.5)	2.6(2.4-2.8)	<0.001
Vegetables (g/d) ^(a)^	173.6(105–245.1)	176.6(104.1-247.2)	162.6(107.4-245.6)	0.302
Fruit (g/d) ^(a)^	193.3(90–310)	191.6(90–313.3)	193.3(86.6-306.5)	0.976
**Iron biomarkers**				
SI (μmol/L serum)	16.8(16.3-17.3)	19.1(18.3-19.7)	14.8(14.2-15.4)	<0.001
SF (μg/L serum) ^(b)^	65.3(65.1-65.5)	114.2(113.9-114.4)	39(38.8-39.2)	<0.001
TFS (%)	33.9(32.7-35)	39.3(37.4-41.1)	28.9(27.7-30.1)	<0.001
**CRP (mg/L serum) **^**(a)**^	1.4(0.5-3.2)	1.4(0.5-2.9)	1.5(0.5-3.4)	0.511

Values are means (IC 95%), percent (IC 95%), (a) median (P25-P75) or (b) geometric mean (IC 95%). Abbreviations: *SFA* saturated fatty acids, *MUFA* monounsaturated fatty acids, *PUFA* polyunsaturated fatty acids, *SI* serum iron, *SF* serum ferritin, *TFS* transferrin saturation, *CRP* C-reactive protein.

### Dietary assessment

The standardized nutrition assessment used in this study has been shown to provide highly reliable nutritional data. Diet was evaluated using the estimated food record method over 3 non-consecutive days, including a weekend or a holiday [8]. The week after the dietary record, a dietary interview was conducted with a nutritionist in order to estimate the portion of food consumed using a book with pictures of pre-weighed food. Two food composition tables were used to calculate daily nutrient intake [9,10].

The interviewers were trained in the evaluation method to standardize their behavior and minimize differences.

### Biochemical variables

Biochemical variables related to iron and oxidative stress were measured with samples of plasma and serum. Blood samples were taken from the antecubital vein after overnight fasting. They were aliquoted and stored at −80°C until analysis.

#### *Serum ferritin (SF), iron (SI), transferrin and transferrin saturation (TFS)*

The analysis of serum ferritin was assessed by immunoassay as described by Gomez et al. [11]. Serum iron and serum transferrin were measured using spectrophotometry (ITC Diagnostics S.A., Barcelona, Spain and Biokit S.A., Barcelona, Spain respectively) with a Beckman Coulter analyzer (Fullerton, California, USA) at the Centro de Investigación Biomédica (CRB of the Hospital Sant Joan de Reus). The transferrin saturation index was calculated.

#### *C-reactive protein*

Since SF can increase in the presence of inflammatory conditions or infections, C-reactive protein (CRP) was measured in serum using the high-sensitivity CRP (hsCRP) technique which is a latex turbidometric immunoassay (Biokit S.A., Barcelona, Spain). CRP was analyzed using a Beckman Coulter analyzer (Fullerton, California, USA)

#### *Lipid oxidation, thiobarbituric acid reactive substances*

Plasma samples were diluted 50-fold in saline before the thiobarbituric acid reactive substances assay (TBARS). TBARS were determined using the Buege and Aust method, but measured using fluorescence at 515 nm (λex) and 548 nm (λem) wavelengths as described by Richard et al. [12]. Malondialdehyde bis (dimethyl-acetal) (MDA) was used as standard and TBARS were expressed as MDA equivalents (nmol/mL).

#### *Plasma total antioxidant capacity, oxygen radical absorbance capacity*

Plasma samples were diluted 500-fold in 75 mM potassium phosphate buffer (pH 7.4) for the analysis of oxygen radical absorbance capacity (ORAC) as previously described by Cao et al. and subsequently modified by Ou et al. [13] with fluorescein as the fluorescent probe. Peroxyl radicals were generated by 2,2’-azobis (2-amidinopropane) dihydrochloride, and fluorescence was monitored at 485 nm (λem) and 538 nm (λem) wavelengths on a Fluoroskan Ascent fluorescence plate reader (Labsystems). 6-hydroxy-2,5,7,8-tetramethylchroman-2-carboxylic acid (Trolox) was used as standard and ORAC was expressed as Trolox equivalents per liter of plasma (mmol TE/L plasma).

### Statistical analyses

The Student t-test and Anova test (or their equivalent non-parametric tests) were used to compare continuous data, and Pearson’s chi-square was used to compare categorical data. Log transformed values were used in the statistical analyses in non-Gaussian distributions.

Normally distributed data were presented in tables as mean ± standard deviation (SD) and non-normally distributed data as geometric mean ± antilog SD or median ± interquartile range (IQR).

To analyze the relationship between antioxidants and pro-oxidants factors obtained from the diet and the biochemical status of iron on lipoprotein oxidation and total antioxidant capacity, multiple linear regression

(MLR) models were applied for the overall sample, as well as in the groups of women and men. We performed two multiple linear regression models for each of the dependent variables (LnTBARS and LnORAC), one with nutritional intake and the other with food group consumption. In the first model (nutrient intake), the following variables were introduced with the “enter” method (independent variables): age, sex, BMI, energy intake (Kcal), SFA, MUFA, PUFA, nutrient intake (vitamin C, vitamin E, β carotene, retinol, non-heme and heme iron), biochemical iron status (serum iron, serum ferritin, transferrin saturation, CRP) and lifestyle factors (alcohol, smoking and physical activity). In the second model (food groups consumption), the variables were: age, sex, BMI, energy intake (Kcal), food consumption (meat, fish, cereals, pulses, vegetables and fruit) biochemical iron status (serum iron, serum ferritin, saturation transferrin, CRP) and lifestyle factors (alcohol, smoking and physical activity). Statistical analyses were performed using the SPSS program for Windows (version 19). In all cases the level of significance was set at p<0.05.

## Results

We selected a total of 1953 individuals randomly, of which 628 were excluded because they did not meet the inclusion criteria. Of the 1325 subjects eligible to participate in the study agreed to enter 817. The main reasons for nonparticipation were work reasons (lack of time), being in drug treatment and the fear of blood collection. We excluded 2 subjects for anemia and iron overload at the same time, signs of hemolytic anemia Additional file 1.

The mean age of the 815 participants in this study was 41 with a standard deviation of 24, and there were slightly more women (52%) than men (48%). The volunteers were stratified by sex and age to ensure a sufficient number of responses from subjects of all ages. Both men and women were divided into four groups: group I, 18 to 29; group II, 30 to 44; group III, 45 to 64; and group IV, 65 to 77 years of age. The distribution of population by age group and sex is summarized in Table 1. Table 1 also gives the socio-demographic characteristics of the subjects with regard to age, lifestyle factors, BMI, education level, dietary energy, nutrient and food intake, biochemical iron status, and CRP. Significant differences between the sexes were found for BMI, alcohol consumption, smoking, physical activity, energy intake (Energy (kcal), SFA, MUFA and PUFA) and daily nutrient intake (vitamin E, β carotene, retinol, non-heme and heme iron). All of the parameters for iron status also showed significant sex differences.

Total antioxidant capacity and lipid peroxidation, which were measured as ORAC and TBARS respectively, are presented in Table 2. The TBARS value increased with age; the youngest group obtained significantly lower TBARS concentrations than the other three groups. No significant differences between the sexes were found.

**Table 2:** Total antioxidant and lipid peroxidation status of the representative sample

	**All participants (n=815)**		***p value***	**Men **^**(1) **^**(n=390)**		***p value***	**Women **^**(2) **^**(n=425)**		***p value***	***p value ***^***(1–2)***^
**ORAC (mmol TE/L plasma)**	7.23	(7.08-7.41)		7.22	(7.01-7.44)		7.25	(7.03-7.48)		0.982
**ORAC age group (years):**										
group I (18–29)	7.21	(6.92-7.51)	1.0 (I-II)	7.61	(7.21-8.04)	1.0 (I-II)	6.89	(6.49-7.3)	1.0 (I-II)	0.347
group II (30–44)	6.77	(6.52-7.02)	1.0 (I-III)	6.64	(6.31-6.98)	1.0 (I-III)	6.89	(6.53-7.26)	1.0 (I-III)	0.687
group III (45–64)	7.51	(7.24-7.79)	1.0 (I-IV)	7.24	(6.85-7.63)	1.0 (I-IV)	7.77	(7.39-8.16)	1.0 (I-IV)	0.471
group IV (65–75)	8.07	(7.5-8.65)	0.817 (II-III)	8.34	(7.61-9.09)	1.0 (II-III)	7.84	(6.99-8.71)	1.0 (II-III)	0.778
			0.358 (II-IV)			0.454 (II-IV)			1.0 (II-IV)	
			1.0 (III-IV)			1.0 (III-IV)			1.0 (III-IV)	
**TBARS (nmol/mL plasma)**	0.93	(0.84-1.03)		0.91	(0.78-1.06)		0.94	(0.81-1.07)		0.244
**TBARS age group (years):**										
group I (18–29)	0.85	(0.66-1.04)	0.004 (I-II)	0.82	(0.54-1.12)	0.037 (I-II)	0.87	(0.62-1.12)	0.167 (I-II)	0.230
group II (30–44)	0.93	(0.78-1.09)	<0.001 (I-III)	0.92	0.92 (0.71-1.14)	<0.001 (I-III)	0.94	(0.73-1.17)	0.04 (I-III)	0.440
group III (45–64)	0.97	(0.8-1.14)	<0.001 (I-IV)	0.97	0.97 (0.73-1.22)	0.034 (I-IV)	0.96	(0.93-1.21)	0.016 (I-IV)	0.852
group IV (65–75)	0.98	(0.72-1.25)	0.771 (II-III)	0.95	0.95 (0.58-1.34)	0.759 (II-III)	1.01	(0.64-1.38)	1.0 (II-III)	0.361
			0.665 (II-IV)			1.0 (II-IV)			0.989 (II-IV)	
			1.0 (III-IV)			1.0 (III-IV)			1.0 (III-IV)	

Values represent geometric mean (IC 95%). *ORAC* oxygen radical absorbance capacity, *TBARS* thiobarbituric acid reactive substances. p-value adjusted with bonferroni correction

Table 3 shows the results that were found to be significant when TBARS was taken as the dependent variable in the regression models. In the first model (nutrient intake), heme iron and age were independently and positively associated with TBARS in all subjects, whereas non-heme iron was negatively associated. In men, age, tobacco and heme iron were positively associated with TBARS. In women, dietary heme iron was negatively associated with TBARS and positively associated with transferrin saturation.

**Table 3:** Association between antioxidant and pro-oxidant diet factors and iron biomarkers on lipid peroxidation (TBARS)

	**Significant variables**	**β coeff**	**SE**	**p-value**	
**Model 1**					
All participants (n=815)	Age, years	0.002	0.001	0.002	R_c_^2^_100_=2.9
	Non-heme iron (mg/d)	−0.012	0.004	0.007	F_3,675_=7.66 p<0.001
	Heme iron (mg/d)	0.012	0.005	0.015	
Men (n=390)	Age, years	0.004	0.001	<0.001	R_c_^2^_100_=6
	Tobacco (cig/d)	0.004	0.002	0.028	F_3,324_=7.94 p<0.001
	Heme iron (mg/d)	0.021	0.007	0.002	
Women (n=425)	Non-heme iron (mg/d)	−0.019	0.006	0.001	R_c_^2^_100_=3.8
	Transferrin saturation (%)	0.003	0.001	0.031	F_2,349_=7.95 p<0.001
**Model 2**					
All participants (n=815)	Age, years	0.002	0.001	0.002	R_c_^2^_100_=2.5
	Fish (g/d)	0.001	0.001	0.004	F_2,697_=9.8 p<0.001
Men (n=390)	Age, years	0.004	0.001	0.001	R_c_^2^_100_=7.3
	Tobacco (cig/d)	0.004	0.002	0.012	F_4,329_=7.5 p<0.001
	Fish (g/d)	0.001	0.001	<0.001	
	Meat (g/d)	0.001	0.001	0.019	
Women (n=425)	Transferrin saturation (%)	0.003	0.001	0.02	R_c_^2^_100_=2
					F_2,363_=4.6 p=0.01

Model 1: Multiple linear regression (MLR) adjusted for age, sex, BMI, energy intake (Kcal, SFA, MUFA, PUFA); nutrient intake (vitamin C, vitamin E, β carotene, retinol, non-heme and heme iron); biochemical iron status (SI, SF, TFS, CRP); lifestyle factors (alcohol, smoking and physical activity). Only variables found to be significant are shown.

Model 2: Multiple linear regression (MLR) adjusted for age, sex, BMI, energy intake (Kcal) food groups consumption (meat, fish, cereals, pulses, vegetables and fruit); biochemical iron status (SI, SF, TFS, CRP); lifestyle factors (alcohol, smoking and physical activity). Only variables found to be significant are shown.

In the second model (with food groups consumption), age and fish consumption were independently and positively associated with TBARS in all subjects. In men, age, tobacco, fish and meat consumption were positively associated with TBARS whereas transferrin saturation was associated in women.

The results that were found to be significant when the dependent variable in the regression model was ORAC are shown in Table 4. In the first model (nutrient intake), Serum ferritin and vitamin C intake were found to be positively associated with ORAC, whereas SFA was negatively associated. In men, CRP and ferritin were positively associated with ORAC, whereas in women the only association was found with serum ferritin.

**Table 4:** Association between antioxidant and pro-oxidant diet factors and iron biomarkers on total antioxidant capacity(ORAC)

	**Significant variables**	**β coeff**	**SE**	**p-value**	
**Model 1**					
All participants (n=815)	Vitamin C (mg/d)	0.009	0.004	0.011	R_c_^2^_100_=3.3
	SFA (g/d)	−0.060	0.023	0.01	F_3,675_=8.79 p<0.001
	Serum ferritin (μg/L)	0.008	0.002	<0.001	
Men (n=390)	Serum ferritin (μg/L)	0.007	0.002	0.002	R_c_^2^_100_=5.8
	CRP (mg/L)	0.303	0.096	0.002	F_2,324_=11.02 p<0.001
Women (n=425)	Serum ferritin (μg/L)	0.023	0.007	0.002	R_c_^2^_100_=2.4
					F_1,350_=9.56 p=0.002
**Model 2**					
All participants (n=815)	Vegetables(g/d)	0.009	0.002	<0.001	R_c_^2^_100_=4.6
	Meat (g/d)	−0.009	0.003	0.002	F_3,696_=12.3 p<0.001
	Serum ferritin (μg/L)	0.008	0.002	<0.001	
Men (n=390)	Serum ferritin (μg/L)	0.007	0.002	0.002	R_c_^2^_100_=5.6
					F_2,331_=10.96 p<0.001
	CRP (mg/L)	0.290	0.095	0.002	
Women (n=425)	Vegetables(g/d)	0.012	0.004	0.001	R_c_^2^_100_=5.3
	Serum ferritin (μg/L)	0.022	0.007	0.002	F_2,663_=11.17 p<0.001

Model 1: Multiple linear regression (MLR) adjusted for age, sex, BMI, energy intake (Kcal, SFA, MUFA, PUFA); nutrient intake (vitamin C, vitamin E, β carotene, retinol, non-heme and heme iron); biochemical iron status (SI, SF, TFS, CRP); lifestyle factors (alcohol, smoking and physical activity). Only variables found to be significant are shown.

Model 2: Multiple linear regression (MLR) adjusted for age, sex, BMI, energy intake (Kcal), food groups consumption (meat, fish, cereals, pulses, vegetables and fruit); biochemical iron status (SI, SF, TFS, CRP); lifestyle factors (alcohol, smoking and physical activity). Only variables found to be significant are shown.

In the second model (food group consumption), serum ferritin and vegetable intake were found positively associated with ORAC, whereas meat intake was negatively associated. In men, we observed again the positive association with CRP and ferritin, and in women we observed a positive association with serum ferritin and vegetables consumption.

## Discussion

Our study was conducted with a sample of 815 randomly selected subjects, which was representative of the adult population according to data from the Statistical Institute of Catalonia (IDESCAT). The publicity strategies used to announce the study and the personalized service provided to each of the participants are directly linked to the high level of participation achieved (61.5%), which is similar to that in other general population studies [14].

Food intake was assessed using 3-day dietary records internationally recognized as a quantitative method for estimating subjects’ regular habits with enough accuracy to permit the inference of partnerships with their nutritional status [15], despite that this method does not take into account seasonal changes. Due to differences in the consumption pattern found in men and women in our sample, we performed all analyzes separated by gender. In 2005, Tur et al. published the results of a food and nutrient intake study in a population similar to ours, and found different behaviors between men and women [16].

The characteristics related to lifestyle (Table 1) also show significant differences in some parameters between both genders. In our sample, men drink more alcohol and smoke more but do more physical exercise than women. In terms of BMI, we found the highest percentage of obesity in men. These results are consistent with the findings of the latest Catalan Health Survey for the adult population (ENCAT). We did not find differences between men and women with regard to education level, which is consistent with the findings published by the statistics office of the Spanish Ministry of Education in 2010 (INE).

Iron levels were determined using levels of transferrin saturation as an indicator of circulating iron, and SF as an indicator of iron stores. According to oxidative theory, elevated levels of either of these pose a health risk [17]. SF is an acute phase reactant and can be increased under inflammatory conditions. Hence, C-reactive protein was analyzed to control the confounding effects of inflammation on serum ferritin.

To determine the parameters for the degree of oxidative stress we used two widely used biomarkers. TBARS assay is inexpensive, fast and very easy to perform. Even though there is controversy regarding the specificity of TBARS toward compounds other than MDA, the fluorometric measurement of TBARS is one of the most commonly used biomarkers for detecting the oxidative damage to lipids, of which MDA is the major compound [18]. The ORAC assay is one of the most reliable methods used to measure the total antioxidant capacity. This assay has frequently been used to provide information about the antioxidant capacity of foods [19]. ORAC is a biomarker that that does not distinguish between the quality and quantity of antioxidants present in plasma; however, ORAC is useful for studies such as ours which aim to determine the overall antioxidant capacity and correlate it with the intake. The use of these two different biomarkers yields a comprehensive view of oxidation in both directions: pro-oxidant and preventive.

In the descriptive analysis, we found that men had a higher energy intake and therefore higher consumption of most of the nutrients studied than women; however, we found no gender difference in the consumption of fruit and vegetables. On the other hand, body iron levels were higher in men than in women, evidence that has been widely connected to higher energy intake, non-menstrual blood loss and hormonal factors.

Our data revealed that the ORAC value was similar in the plasma of the younger and the older subjects and that there were no differences between the ORAC values of men and women. These gender results are in agreement with previous studies [20], although some authors have found differences between age groups [21]. The use of this biomarker to determine the total antioxidant level in plasma has only been used to compare pathology groups with healthy controls, but studies assessing healthy subjects separated by age and sex are scarce. The role of oxidative damage in normal aging has been substantiated in studies with experimental animals, but there is limited evidence in humans. In our study, TBARS levels were found to increase with age, which is consistent with the results of other researchers [22]. Furthermore, our results support the “free radical theory of aging” proposed in 1956 by Harman based on the accumulation of oxidatively damaged molecules.

Voss et al. reviewed the behavior of oxidative stress, the glutathione system and antioxidant systems related to diet and lifestyle in relation to age [18]. They predicted an increase in oxidative stress, a decline in the glutathione system, and the lifetime preservation of antioxidant systems related to diet. In this respect, our results are consistent with this reasoning, as we found an increase in TBARS and similar ORAC values at different ages.

To study the association between diet and biochemical iron levels with oxidative stress, we used dietary factors associated with oxidation or dietary iron absorption, adjusting for confounding factors.

There are two forms of dietary iron: heme and non-heme. Iron in meat, fish and poultry is found in a chemical structure known as heme. The body absorbs heme iron very efficiently. Iron in vegetables and legumes is arranged in a different chemical structure called non-heme iron. Non-heme iron is not as well absorbed as heme iron [23]. As shown in Table 3, dietary heme and non-heme iron have differing relationships with oxidative stress. Non-heme iron is related to a decrease in oxidative damage as measured by TBARS, whereas the opposite is true of heme iron. These results reflect the effect of diet on oxidative stress, i.e. the consumption of products rich in non-heme iron (vegetables) linked to a diet rich in antioxidants, and the consumption of heme iron (from meat and fish) associated with a pro-oxidant diet. The possible pro-oxidant properties of heme iron have been the subject of study in the past [24] and a close relationship was identified between heme iron intake and the risk of mortality from cardiovascular disease, lung cancer or upper digestive tract cancer and general mortality [25]. In contrast, non-heme iron intake is negatively associated with this peroxidation. This may be because non-heme iron is found mainly in leafy green vegetables, fruit and vegetables, which are also a good source of vitamins and antioxidants which may decelerate the negative effect of iron on human health.

Comparing the results for men and women separately, it becomes clear that in men smoking is associated with oxidative stress, a connection which is widely known. It also seems that increased oxidative stress in women is related to higher transferrin levels. Iron can damage tissues by catalyzing the conversion of hydrogen peroxide to free-radical ions which attack cellular membranes, proteins, and DNA. Proteins sequester iron to reduce this threat. Iron ions circulate bound to plasma transferrin and accumulate within the cells in the form of ferritin [26]. The presence of free iron has been associated with low concentrations of transferrin accompanied by an increase in its saturation in an attempt to prevent the accumulation of that free iron in plasma. Many studies have linked high and even moderate levels of iron with many chronic and common diseases in the population, such as cardiovascular disease and cancer [27]. According to these studies, iron, as an oxidant mineral, could lead to increased oxidative stress and therefore promote all types of diseases related to oxidation. Given that the participants in this study are not patients but members of the general population, we believe that the increased transferrin saturation may be a reflection of higher levels of free iron and therefore of increased oxidative stress (TBARS).

When the same MLR models were applied to look at antioxidant capacity, many points of similarity with the previous study emerged. First, a diet rich in saturated fatty acids, which are a source of oxidizable lipids, is related to a decrease in antioxidant capacity and a diet rich in vegetables, which is a source of antioxidants, and vitamin C intake are related to an increase in ORAC. Both results are consistent with findings in the existing literature [28]. This increase in antioxidant capacity along with increased SF supports the hypothesis posed by Juckett et al. [29]. They suggested that ferritin may act as a protective mechanism against oxidative processes, and that oxidative stress induces the synthesis of ferritin to capture the oxidized iron, and thus preventing toxicity.

The positive relationship between increased iron storage and antioxidant status does not differ between men and women. However, in men ORAC increases significantly with C-reactive protein. Since CRP is a marker of inflammation, this could indicate a possible mechanism for defending the body against oxidative stress in situations such as inflammation. Peairs et al. [30], in an intervention study with a low-carbohydrate, weight-loss diet, found a significant decrease in some indicators of inflammation (e.g. MCP-1) but not others (e.g. IL-6, CRP) within seven days. Recently, Floegel et al. [31] concluded that the antioxidant intake from supplements may have additional beneficial effects on plasma Hcy but not on serum CRP concentrations in the US population. Therefore, the relationship between antioxidant status, CRP and SF is not clear and requires further research.

As mentioned earlier, an increase in the consumption of vegetables and vitamin C is associated with improved antioxidant capacity, but a regression analysis separating subjects by gender revealed that this improvement is exclusive to women. Although no differences were recorded in vegetable consumption between men and women, the significant increase of some antioxidants like beta carotene and vitamin C in women might indicate that the consumption of vegetables by gender does not differ in quantity but may differ in quality.

## Conclusions

In conclusion, it is evident from this study that oxidative stress is related to aging regardless of sex, but no alteration in antioxidant capacity is related to age. Moreover, we observed an association between diet and biochemical iron levels with the antioxidant and pro-oxidant status of the organism in our general population. The highest intake of dietary non-heme iron, vegetables and vitamin C appear to be protective against oxidation while the highest intake of dietary heme iron from meat and fish and saturated fatty acids are associated with increased oxidative stress. Also, the high levels of circulating iron measured by transferrin saturation are associated with increased oxidative stress in women however the association with the higher levels of serum ferritin is controversial and needs to be studied.

## Abbreviations

(TBARS): Thiobarbituric acid reactive substances; (ORAC): Oxygen radical absorbance capacity; (SF): Serum ferritin; (SI): Serum iron; (TFS): Transferrin saturation; (CRP): C-reactive protein; (hsCRP): High-sensitivity CRP; (MDA): Malondialdehyde bis(dimethyl-acetal); (Trolox): 6-hydroxy-2,5,7,8-tetramethylchroman-2-carboxylic acid.

## Competing interests

The authors declare that they have no competing interests.

## Authors’ contributions

The authors’ contributions to manuscript were as follows: VA: study concept and design; VA and MG: conducted research; MRN, MR, NA and BR: acquisition of data; MR, NA, MG and VA: analysis and interpretation of data; MR and NA: statistical analysis; MR and NA: drafting of the manuscript; VA had primary responsibility for final content. All authors read and approved the final manuscript.

## Supplementary Material

Additional file 1 Flow diagram. Click here for file
