# Supplementary material for: Diet, iron biomarkers and oxidative stress in a representative sample of Mediterranean population
Source: Nutr J. 2013 Jul 16;12:102. doi: 10.1186/1475-2891-12-102 (PMC3847655; doi:10.1186/1475-2891-12-102)
Supplement: Additional file 1 — Flow diagram. [file 1475-2891-12-102-S1.doc]

**Flow Diagram**

Assessed for eligibility (n=1953)

Excluded (n=1136)

♦  Not meeting inclusion criteria (n=628 )

♦  Declined to participate (n=508 )

Analysed (n=815)

♦  Excluded from analysis (hemolytic anemia suspected) (n=2 )
